# Years of life lost due to premature death and their trends in people with malignant neoplasm of female genital organs in Shanghai, China during 1995–2018: a population based study

**DOI:** 10.1186/s12889-020-09593-6

**Published:** 2020-10-01

**Authors:** Zheng Luo, Yuan He, Guifen Ma, Yang Deng, Yichen Chen, Yi Zhou, Xiaoyun Xu, Xiaopan Li, Yan Du

**Affiliations:** 1 Shanghai University of Medicine & Health Sciences Affiliated Zhoupu Hospital, No. 1500 Zhouyuan Rd., Pudong New Area, Shanghai, 201318 China; 2 Obstetrics and Gynecology Hospital of Fudan University, 419 Fangxie Road, Shanghai, 200011 China; 3 Department of Radiotherapy, Zhongshan Hospital, Fudan University, Shanghai, 200032 China; 4 School of Public Health, Shandong First Medical University & Shandong Academy of Medical Sciences, Tai’an, 271016 Shandong China; 5 Center for Disease Control and Prevention, No. 3039 Zhangyang Rd., Pudong New Area, Shanghai, 200136 China; 6 Fudan University Pudong Institute of Preventive Medicine, Pudong New Area, Shanghai, 200136 China

**Keywords:** Malignant neoplasm of female genital organs, Mortality, Years of life lost, Trend analysis, Decomposition method

## Abstract

**Background:**

The increasing aging population has been posing a significant challenge to disease burden in developing countries. In particular, the contribution of population aging to and long term changes of disease burden of malignant neoplasm of female genital organs (MNFGO) have not been quantitatively demonstrated.

**Methods:**

Data were collected from the Shanghai Vital Statistics System of Pudong New Area (PNA). Crude mortality rate (CMR), age-standardized mortality rate by Segi’s world standard population (ASMRW), and years of life lost (YLL) of MNFGO as the underlying cause of death in age and pathology types from 1995 to 2018 were calculated. The joinpoint regression was used to estimate the trends of those rates by identifying the annual percent changes (APCs), and the decomposition method was used to calculate the increased rates and the contribution resulting from demographic and non-demographic factors.

**Results:**

From 1995 to 2018, a total of 2869 MNFGO-specific deaths were reported in PNA, accounting for 0.64% of the total deaths. The CMR and ASMRW of MNFGO were 9.23/10^5^ person-years and 4.80/10^5^ person-years, respectively. Ovary cancer was the most common cause of MNFGO death, accounting for 43.9% (1260/2869) of all MNFGO death. Other common causes of MNFGO death included cervix uteri cancer, uterus unspecified cancer, and corpus uteri cancer. With the increase of age, the mortality rate of MNFGO in residents had shown an upward trend ([APC (95%CI) = 3.46 (2.74, 4.18), *P* < 0.001)] for each five-year age group from 0 to 4 to 85+ years. From 1995 to 2018, YLL of MNFGO in Shanghai PNA was 42,152.82 years, and the rate of YLL was 135.56 /10^5^. The top three MNFGO types in YLLs were ovary cancer, cervix uteri cancer and uterus unspecified cancer. Demographic factors contributed significantly to the upward trends of CMR, ASMRW, and YLL rates of MNFGO.

**Conclusion:**

With aggravated population aging in Shanghai, MNFGO is and will continue to be a serious threat to women’s health. More precise and effective prevention strategies are needed to target high risk population, to achieve efficient health resource allocation and to improve women’s health in particular.

## Background

Cancer has become the leading cause of death since 2010 in China [1]. Gynecological cancer is one of the most common cancer types in females. Particularly, the morbidity and mortality of gynecological malignancies are on the rise [1]. This increased health burden may be partially attributed to demographic changes such as aging, as well as lifestyle changes associated with rapid economic development [2]. China has undergone fast demographic and epidemiological changes in the past few decades. Shanghai has been the forerunner of urbanization and socioeconomic development in China [2]. Shanghai is a metropolitan city located in the east coast of China, and the geographical and population characteristics make it a representative and reliable sample that can reflect the disease burden and mortality trend of inland China in the next 20 years. Shanghai Pudong New Area (PNA) covers an area of 1210.41 km^2^ (467.34 mile^2^) with a resident population of 5.5 million, among which more than 3.0 million are registered permanent residents, according to the 2018 Household Registry [3]. PNA is the district with the largest geographic area and accounts for one fifth of the total population in Shanghai [3]. PNA has been oriented as a national economic and technological development zone since early 1990s, consisting of a mixture of urban, suburban, and rural geographic areas [4]. It is a typical sample of China’s reformation and urbanization. Hence, it is the microcosm of China’s reformation and a good representative of Shanghai [5].

Statistics have shown that cancer is expected to become the single most important barrier to increasing life expectancy in the world [6]. Age is a major risk factor of cancer. The increasing aging population and rapid agglomeration of population to Shanghai brings enormous pressure to public health and environment [7]. The severity of cancer is often measured in number of deaths. However, the number of deaths alone does not reflect the complete burden put on the society, therefore the number of years of life lost (YLL) depending on the age at death and the number of deaths at each age may be a more appropriate indicator of impact on society and cancer prevention [8]. Monitoring levels and trends in premature mortality is crucial to understand how societies can address prominent sources of early death [9]. In particular, the contribution of population aging to and long term changes of disease burden of malignant neoplasm of female genital organs (MNFGO) have not been quantitatively demonstrated. In the current study, we analyzed the mortality and YLL due to premature death and their trends in people with NMFGO in PNA of Shanghai, China during 1995 to 2018.

## Methods

### Data source

The data of cancer death were collected from the Mortality Registration System of Shanghai PNA [4]. The population data were provided by the Statistics Bureau and the Public Security Bureau of PNA [4, 10]. The population data are from the year 1995 to 2018, which are all the years with complete population data available. There are a total of 31,094,930 person-years for women from 1995 to 2018. Figure S1 shows the detailed age composition of Shanghai PNA population from 1995 to 2018. The Mortality Registration System of PNA covers medical institutions of all levels and data are checked against local population registry on a monthly basis [4]. To ensure the completeness of the registration system to the maximum extent, periodic evaluations, data cleaning and compilation have been done at both the county and provincial levels according to standard guidelines and have been validated [4]. The per capita Gross Domestic Product (GDP) of Shanghai and Shanghai PNA were collected from Shanghai Municipal Bureau of Statistics (http://tjj.sh.gov.cn/) and Shanghai PNA Bureau of Statistics (http://www.pudong.gov.cn).

Deaths from MNFGO (C51-C58) including vulva cancer (C51), vagina cancer (C52), cervix uteri cancer (C53), corpus uteri cancer (C54), uterus unspecified cancer (C55), ovary cancer (C56), other female genital organs cancer (C57) and placenta cancer (C58), were classified by the underlying cause of deaths according to the International Classification of Diseases 10th version (ICD-10) [11]. Since the data covered a long time span of 23 years, data for 1995–2001 was coded based on the International Classification of Diseases 9th version (ICD-9) and was recoded to ICD-10. Detailed coding, conversion and verification process was described previously [4]. All causes of death were coded by rigorously trained clinicians, and each record was further verified by local Center for Disease Control and Prevention (CDC). Percentages of cancers morphology verified (MV%) and death certificate only (DCO%) showed overall data quality is good.

### Statistical analyses

The crude mortality rates (CMR) were calculated as the total number of deaths each year divided by the corresponding annual average population in PNA and expressed as per 100,000 (/10^5^) population. The rates were age-standardized by Segi’s world standard population (ASMRW) in gender. YLL was calculated according to the original method of Murray and Lopez [12]. To calculate YLLs due to death, the number of deaths was multiplied by standard life expectancy for each age group, and summed for all age groups. The equation used to calculate YLL is presented below, with a 0.03 age-weighting rate and 0.04 time-discount rate [13]:

$$ \mathrm{YLL}={\mathrm{KCe}}^{\mathrm{ra}}/{\left(\mathrm{r}+\upbeta \right)}^2\left\{{\mathrm{e}}^{\hbox{-} \left(\mathrm{r}+\upbeta \right)\left(\mathrm{L}+\mathrm{a}\right)}\left[\hbox{-} \left(\mathrm{r}+\upbeta \right)\left(\mathrm{L}+\mathrm{a}\right)\hbox{-} 1\right]\hbox{-} \mathrm{e}{\hbox{-}}^{\left(\mathrm{r}+\upbeta \right)\mathrm{a}}\left[\hbox{-} \left(\mathrm{r}+\upbeta \right)\mathrm{a}\hbox{-} 1\right]\right\}+\left(1\hbox{-} \mathrm{k}\right)/\mathrm{r}\ast \left(1\hbox{-} {\mathrm{e}}^{\hbox{-} \mathrm{rL}}\right) $$

where: r = discount rate, β = age-specific weight parameter, K = use of age-specific weight (using age-weight, applied 1; not using age-weight, applied 0), C = constant, a = age at death, L = life expectancy at death. The calculation of YLL was performed using the World Health Organization (WHO) template [14]. Rates of YLL due to MNFGO were calculated and shown as per 100,000 (/10^5^) [13].

To calculate the disease burden of premature death in 2869 women died of MNFGO from 1995 to 2018, cancer registration data from Shanghai PNA and the causes of mortality data by The Mortality Registration System of PNA for 447,861 total deaths during the same time period were linked via personal identification number.

Temporal trends of CMR, ASMRW, and rate of YLL were calculated using Joinpoint Regression Program 4.3.1.0 (National Cancer Institute, Bethesda, MD, USA) and expressed as an annual percent change (APC) with corresponding 95% confidence interval (95% CI). Z test was employed to assess whether the APC was statistically different from zero. Terms of “increase” or “decrease” were used to describe statistically significant (*P* < 0.05) APC, while “stable” was used for not statistically significant trends.

Age was classified into 7 groups: 0–14 year, 15–29 years, 30–44 years, 45–59 years, 60–69 years, 70–79 years and 80+ years. Age-specific CMRs were calculated for each age group. The increased mortality rates of each period in 3 years from 1998 to 2018, compared with the data during 1995–1997 or the period before it, caused by demographic and non-demographic factors were estimated by the decomposition method, in which mortality rates were calculated and compared for each five-year age group, from 0 to 4 to 85+ years [15]. All statistical analyses were conducted using SPSS 21.0 (SPSS, Inc., Chicago, IL) and R (version 3.4.3). *P* value of < 0.05 was considered as statistically significant.

## Results

### Number of deaths and mortality rates of MNFGO

There were 447,861 total deaths including 211,596 female deaths in Shanghai PNA from 1995 to 2018. During the same period, a total of 2869 MNFGO-specific deaths were reported in PNA, accounting for 0.64% of the total deaths and 1.36% of female deaths. The CMR and ASMRW of MNFGO were 9.23/10^5^ person-years and 4.80/10^5^ person-years, respectively. The average age at death from MNFGO in PNA was 65.08 ± 14.59 years old, and the median age at death from MNFGO in PNA was 64.92 years old. Ovary cancer (C56) was the most common cause of MNFGO death, accounting for 43.9% (1260/2869) of all MNFGO death. Other common causes of MNFGO death included cervix uteri cancer (C53), uterus unspecified cancer (C55), and corpus uteri cancer (C54). CMR, ASMRW, average age and median age at death in different types of MNFGO are presented in Table 1.

**Table 1:** Baseline characteristics of deaths from MNFGO in Shanghai PNA, 1995–2018

Site	Deaths (N, %)	Age in years (Mean ± SD)	Age in years (Median)	CMR (/10^**5**^)	ASMRW (/10^**5**^)	YLL (years)	YLL rate (/10^**5**^)
**Vulva cancer (C51)**	71 (2.47)	77.50 ± 11.32	79.12	0.23	0.09	661.58	2.13
**Vagina cancer (C52)**	40 (1.39)	69.15 ± 12.58	70.43	0.13	0.06	518.42	1.67
**Cervix uteri cancer (C53)**	677 (23.60)	63.51 ± 16.86	62.59	2.18	1.14	10,272.51	33.04
**Corpus uteri cancer (C54)**	293 (10.21)	67.72 ± 11.88	67.65	0.94	0.47	4007.61	12.89
**Uterus unspecified cancer (C55)**	471 (16.42)	69.41 ± 14.14	70.38	1.15	0.72	6075.53	19.54
**Ovary cancer (C56)**	1260 (43.92)	62.86 ± 13.54	62.08	4.05	2.23	19,763.83	63.56
**Other female genital organs cancer (C57)**	56 (1.95)	65.56 ± 10.03	66.01	0.18	0.10	826.37	2.66
**Placenta cancer (C58)**	1 (0.03)	28.17	28.17	0.00	0.00	26.98	0.09
**Total**	2869 (100.00)	65.08 ± 14.59	64.92	9.23	4.8	42,152.82	135.56

*ASMRW* Age-standardized mortality rate by Segi’s world standard population, *CMR* Crude mortality rate, *PNA* Pudong New Area, *YLL* Years of life lost, *MNFGO* Malignant neoplasm of female genital organs

In 2008, Shanghai PNA entered the aging society (14.21%); and in 2018, Shanghai has entered the super-aging society (20.81%) [16]. The proportion of the ≥65 years age group in PNA had been increasing significantly (APC = 3.05, 95% CI = 2.80%; 3.31%, *P* < 0.001), and the proportion of the ≥65 years age group in the female population also showed a significant upward tread (APC = 2.65, 95% CI = 2.40%; 2.90%, *P* < 0.001) (Figure S2). From 1995 to 2018, the elderly population over 60 years old was 1772, accounting for 61.76% of the total population. With the increase of age, the mortality rate of MNFGO in residents had shown an upward trend (APC = 3.46, 95% CI = 2.74; 4.18, *P* < 0.001) for each five-year age group from 0 to 4 to 85+ years. The MNFGO mortality rates of 0–14 y, 15–29 y, 30–44 y, 45–59 y, 60–69 y, 70–79 y and 80+ y age groups were 0.03/10^5^ person-years, 0.52/10^5^ person-years, 2.86/10^5^ person-years, 11.43/10^5^ person-years, 18.50/10^5^ person-years, 27.20/10^5^ person-years, and 41.51/10^5^ person-years, respectively (Table 2).

**Table 2:** Number of deaths, CMR, YLLs and rates of YLL due to MNFGO in different age groups in Shanghai PNA, 1995–2018

Age group	Deaths (N)	Proportion (%)	CMR (/10^**5**^)	YLL (years)	YLL rate (/10^**5**^)
**0–14 y**	1	0.03	0.03	29.30	1.16
**15–29 y**	29	1.01	0.52	793.35	14.25
**30–44 y**	214	7.46	2.86	5173.21	69.20
**45–59 y**	853	29.73	11.43	17,084.52	228.85
**60–69 y**	658	22.93	18.50	10,059.13	282.85
**70–79 y**	586	20.43	27.20	6008.48	278.92
**80+ y**	528	18.40	41.51	3004.83	236.25
**Total**	2869	100.00	9.23	42,152.82	135.56

*CMR* Crude mortality rate, *MNFGO* Malignant neoplasm of female genital organs, *PNA* Pudong New Area, *YLL* Years of life lost

Ovary cancer was the leading cause of MNFGO death among all age groups except for age group 30–44 y, where cervix uteri cancer was the leading cause of MNFGO death. Furthermore, the top three causes of death in the 80 + y group were very close, which included ovary cancer (*n* = 150), cervix uteri cancer (*n* = 149) and uterus unspecified cancer (*n* = 120). The number and proportion of different causes of MNFGO death in each age group are presented in Table S1.

### Burden of premature death due to MNFGO

From 1995 to 2018, YLL of MNFGO in Shanghai PNA was 42,152.82 years, and the rate of YLL was 135.56/10^5^. The top three MNFGO types in YLLs were ovary cancer, cervix uteri cancer and uterus unspecified cancer, contributed to a loss of 19,763.83 years (46.89%), 10,272.51 years (24.37%), and 6075.53 years (14.41%), respectively (Table 1). As for age groups, the top three in YLLs were 45–59 y,60–69 y and 70–79 y, which were 17,084.52 years (40.53%), 10,059.13 years (23.86%) and 6008.48 years (14.25%), respectively; while the top three age groups in rate of YLL were 60–69 y,70–79 y and 80+ y, which were 282.85/10^5^, 278.92/10^5^ and 236.25/10^5^, respectively. Table 1 presents the YLLs and rates of YLL in different MNFGO types, and Table 2 in different age groups.

### Trends of different MNFGO rates

**Fig. 1:**
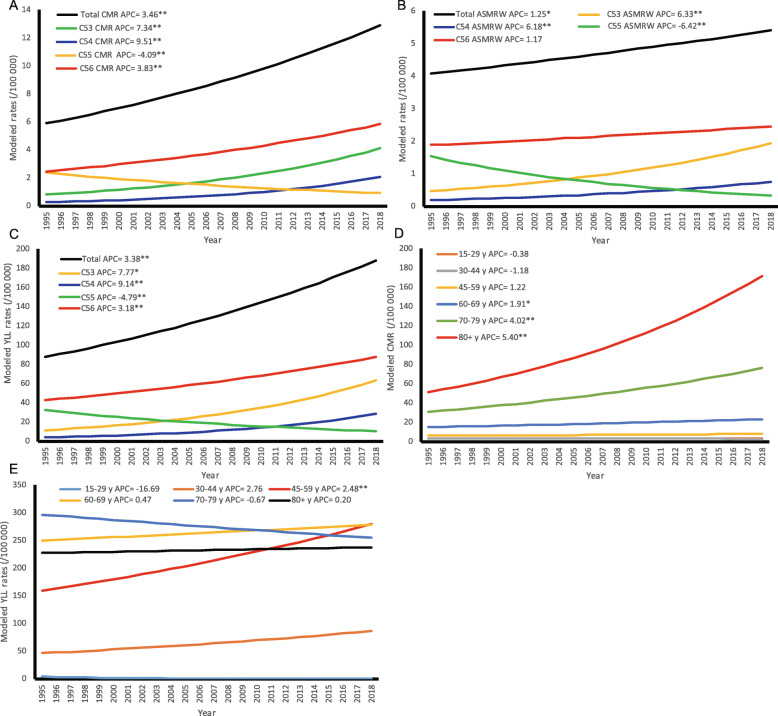
The trends of CMR, ASMRW and YLL rate of persons with underlying cause of MNFGO death by pathology types and age groups in Shanghai Pudong New Area from 1995 to 2018. **a**, CMR of pathology types; **b**, ASMRW of pathology types; **c**, YLL rate of pathology types; **d**, CMR of age groups; **e**, YLL rate of age groups. Abbreviations: ASMRW, age-standardized mortality rate by Segi’s world standard population (per 100,000); CMR, crude mortality rate (per 100,000); YLL, years of life lost (per 100,000). * *P*-Value < 0.05; ** *P*-Value < 0.001

In 1995–2018, the modeled CMR, ASMRW and YLL rate in different MNFGO types and age groups were shown in Fig. 1a-c and Fig. 1d-e, respectively. The observed CMR, ASMRW and YLL rate of all MNFGO in different cancer types and age groups were shown in Figure S3 A-E. The observed values of CMR, ASMRW, YLL and YLL rate and the top three cancer types (cervix uteri cancer, corpus uteri cancer, and ovary cancer) have increased since the year of 1995, and peaked in the year of 2018 (Table S2). While the above observed values have decreased over the years for uterus unspecified cancer (Table S2).

Joinpoint trend analysis of the CMR, ASMRW, YLL rate of different MNFGO cancer types are shown in Table 3 and Fig. 1a-c, and in each age group are shown in Table 4 and Fig. 1d-e. Significant upward trends of CMR were observed in cervix uteri cancer, corpus uteri cancer, and ovary cancer, during the 23 years. Between 1995 and 2018, the value of CMR increased in each year on average by 7.34% (95% CI = 5.59; 9.12, *P* < 0.001) for cervix uteri cancer, 9.51% (95% CI = 7.73; 11.33, P < 0.001) for corpus uteri cancer, 3.83% (95% CI = 2.63; 5.03, *P* < 0.001) for ovary cancer, respectively. However, the CMR value of uterus unspecified cancer decreased by 4.09% per year (95% CI = − 5.33; − 2.84, *P* < 0.001) during the study period. There were significant upward trends from 1995 to 2018 in ASMRW in cervix uteri cancer (6.33% per year, 95% CI = 4.41; 8.27, *P* < 0.001), and corpus uteri cancer (6.18% per year, 95% CI = 4.32; 8.07, *P* < 0.001). While for uterus unspecified cancer, the value of ASMRW decreased in each year on average by 6.42% (95% CI = − 7.87; − 4.94, *P* < 0.001) during the same time period. Furthermore, significant upward trends of YLL rate were observed in cervix uteri cancer (7.77% per year, 95% CI = 5.82; 9.75, *P* < 0.001), corpus uteri cancer (9.14% per year, 95% CI = 7.55; 10.76, *P* < 0.001), and ovary cancer (3.18% per year, 95% CI = 1.89; 4.49, *P* < 0.001) from 1995 to 2018. While for uterus unspecified cancer, YYL rate decreased in each year on average by 4.79% (95% CI = − 6.27; − 3.30, *P* < 0.001).

**Table 3:** Annual percent change of CMR, ASMRW, and YLL rate in different MNFGO cancer types in Shanghai PNA, 1995–2018

Cancer types	APC (95% CI) (%)		
	**CMR**	**ASMRW**	**YLL rate**
**Cervix uteri cancer (C53)**	7.34 (5.59, 9.12)**	6.33 (4.41, 8.27)**	7.77 (5.82, 9.75)**
**Corpus uteri cancer (C54)**	9.51 (7.73, 11.33)**	6.18 (4.32, 8.07)**	9.14 (7.55,10.76)**
**Uterus unspecified cancer (C55)**	−4.09(−5.33, −2.84)**	−6.42(−7.87, −4.94)**	−4.79(−6.27, −3.30)**
**Ovary cancer (C56)**	3.83 (2.63, 5.03)**	1.17(− 0.06, 2.43)	3.18 (1.89, 4.49)**

**P < 0.001

*95% CI* 95% confidence interval, *APC* Annual percent change, *ASMRW* Age-standardized mortality rate by Segi’s world standard population, *CMR* Crude mortality rate, *MNFGO* Malignant neoplasm of female genital organs, *PNA* Pudong New Area, *YLL* Years of life lost

**Table 4:** Annual percent change of CMR, ASMRW, and YLL rate of MNFGO in different age groups in Shanghai PNA, 1995–2018

Age Group	APC (95% CI) (%)		
	CMR	ASMRW	YLL rate
**15–29 y**	−0.38(−2.30, 1.59)	/	−16.69(−45.42, 27.18)
**30–44 y**	−1.18(−3.36, 1.05)	/	2.76(−0.14,5.75)
**45–59 y**	1.22(− 0.14,2.60)	/	2.48 (1.28,3.69)**
**60–69 y**	1.91 (0.86, 2.97)*	/	0.47(−1.04,2.00)
**70–79 y**	4.02 (3.03,5.02)**	/	−0.67(−2.37,1.07)
**80+ y**	5.40 (4.27,6.55)**	/	0.20(−1.30, 1.71)
**Total**	3.46 (2.74, 4.18)**	1.25 (0.35, 2.15)*	3.38 (2.62,4.14)**

*P < 0.01, **P < 0.001

*95% CI* 95% confidence interval, *APC* Annual percent change, *ASMRW* Age-standardized mortality rate by Segi’s world standard population, *CMR* Crude mortality rate, *MNFGO* Malignant neoplasm of female genital organs, *PNA* Pudong New Area, *YLL* Years of life lost

From 1995 to 2018, the CMR values increased in each year on average by 1.91% (95% CI = 0.86; 2.97, *P* < 0.05) for age group 60–69 y, 4.02% (95% CI = 3.03; 5.02, *P* < 0.001) for age group 70–79 y, and 5.40% (95% CI = 4.27; 6.55, *P* < 0.001) for age group 80+ y. For the total population, the CMR values increased by 3.46% per year (95% CI = 2.74; 4.18, *P* < 0.001) between 1995 and 2018. The YLL rate increased by 2.48% per year (95% CI = 1.28; 3.69, *P* < 0.001) for age group 45–59 y, and increased in each year on average by 3.38% per year (95% CI = 2.62; 4.14, P < 0.001) for the total population (Table 4).

### Quantitative impact of demographic and non-demographic factors on MNFGO

**Fig. 2:**
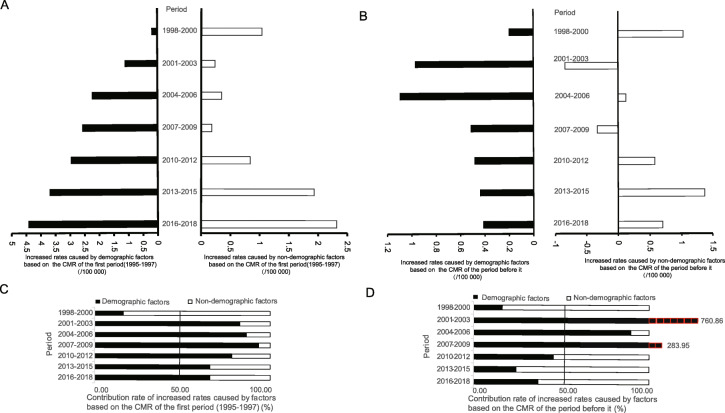
The increased rates caused by demographic and non- demographic factors and their contribution rates from 1998 to 2018 in Shanghai Pudong New Area. **a** & **c**, based on the first period; **b** & **d**, based on the period before it

Based on the CMR of MNFGO in 1995–1997, there were significant upward trends from 1998 to 2018 in difference of mortality (APC = 34.75, 95%CI = 27.25; 42.71, *P* < 0.001), and there were significant upward trends in increased rates caused by demographic factors (APC = 53.37, 95% CI = 15.56; 103.55, *P* = 0.012), but there were no significant upward trends in increased rates caused by non-demographic factors (APC = 30.69, 95% CI = − 15.85; 102.97, *P* = 0.179) (Fig. 2a and Table S3). From 2001 to 2018, the contribution of increased values of CMR caused by demographic factors was over 50%, as shown in Fig. 2c.

Based on the CMR of the period before it, there were no significant upward trends in difference of mortality or increased rates (all *P* > 0.05) (Fig. 2b and Table S3). The contribution of increased values of CMR based on the period before it for three consecutive time periods of 2001–2003, 2004–2006, and 2007–2009 was 760.86, 89.66, and 283.95%, respectively, which were all exceeding 50%, as shown in Fig. 2d and Table S3.

## Discussion

The top three causes of MNFGO deaths in Shanghai PNA during the period of 1995 to 2018 were ovary cancer (C56), cervix uteri cancer (C53), and uterus unspecified cancer (C55). Together, these three cancer types account for more than 80% of the total MNFGO death in PNA of Shanghai. Ovary cancer, cervix uteri cancer, and uterus unspecified cancer were also the top three in YLLs in our study. In general, there were significant upward trends of CMR as well as ASMRW in the total population. Similar to our data, an increasing trend in mortality was observed for the above three types of cancer (cervix, uterine corpus and ovary cancer) during 2000–2011 in China [1]. MNFGO poses great threats to women’s health, and especially affects reproductive health in women of childbearing age. The Global Burden of Disease (GBD) Cancer Collaboration study reported that the estimated age-standardized death rate (ASDR) for cervical cancer, ovarian cancer, and uterine cancer was 6.1/10^5^ [95% uncertainty interval (UI) = 5.7/10^5^; 6.4/10^5^], 4.1/10^5^ (95% UI = 4.0/10^5^; 4.3/10^5^), and 2.0/10^5^ (95% UI = 1.9/10^5^; 2.0/10^5^), respectively. Cervical cancer, ovarian cancer, and uterine cancer ranked 9th, 14th, and 24th respectively by absolute YLLs among both sexes between 2007 and 2017 [17].

Our study showed that ovary cancer surpassed cervical cancer as the leading cause of MNFGO death in Shanghai PNA, which is different from the GBD data [17]. Ovary cancer is a fatal disease with a very poor prognosis [18]. Despite advancement in recent decades, the 5-year survival rate remains around 45% in the United States [19]. More importantly, there was an increasing trend of ovary cancer deaths in Shanghai PNA from 1995 to 2018. There is yet no reliable screening method for ovary cancer. Epidemiological and mechanism studies are needed to elucidate ovary cancer etiology, and to develop better treatment and eventually improve prognosis.

The GBD study reported that cervical cancer was the most common cause of cancer deaths for women in 39 countries [17], and the national data also reported that cervix uteri cancer was the leading cause of gynecological cancer death in China [1]. Contrary to the national data, cervix uteri cancer ranked as the second common MNFGO death in PNA of Shanghai, probably because of better availability of screening test and early treatment. Similar to the GBD study reporting deaths from cervix cancer increased by 19% (95% UI: 13%; 23%) globally between 2007 and 2017, our results also showed an increased trend of cervix uteri cancer death during the same period of time. Currently, even though HPV vaccination program will prevent a proportion of cervix uteri cancer, a significant amount of women aged above 26 are not eligible for receiving the HPV vaccine in mainland China [20]. More importantly, cervix uteri cancer surpassed ovary cancer and ranked as the leading cause of MNFGO death in PNA women of age group 30–44 y. The premature death of this age group of women not only affects the individuals, but also places great burden on the families and society as well. Therefore, the implementation of community based health education and early detection programs targeting the key population are of crucial importance.

The national statistics showed that the CMR of corpus uteri cancer (C54–55) was 2.97/10^5^ from 2008 to 2012, making it the 13th leading cause of cancer-related death in females which was much higher than the GBD ranking (24th) [17, 21]. Our data showed slightly lower CMR (2.09/10^5^) compared to the national data during the same period, probably due to more advanced technology and comprehensive health care system in Shanghai. Risk factors of corpus uteri cancer represented by endometrial cancer cases (C54) include hormones, overweight or obesity, meat intake or physical inactivity [22]. With rapid socioeconomic development, increasing rate of urbanization, changes in lifestyle and dietary structure of the Shanghai population, corpus uteri cancer is likely to continue to be a serious threat to women’s health.

Like other types of cancer, our data showed that demographic factors contributed significantly (over 50% from 2001 to 2018) to the increased values of the CMR of MNFGO. With aggravated population aging in Shanghai, MNFGO are and will continue to be a huge health burden and seriously endanger women’s health. On the contrary, no significant trends of increased rates of MNFGO were observed for non-demographic factors in our data. Our data showed that per capita GDP in Shanghai (APC = 2.32, 95% CI = 11.30; 13.35, *P* < 0.001) as well as PNA (APC = 10.90, 95% CI = 10.38; 11.43, *P* < 0.001) had been increasing significantly from 1995 to 2018 (Figure S2). Economic development such as increased GDP improves women’s health in general; however, some factors related to developed economy such as lifestyle, diet, and reproductive changes may also increase the risk of reproductive system diseases. Hence, community-based cancer prevention and early detection programs should be tailored to address special needs in target populations.

Monitoring levels and trends in premature mortality is crucial to understand how societies can address prominent sources of early death. Chinese government released the blueprint guide of “Healthy China 2030”, in which an official goal was set to reduce premature mortality of major non-communicable diseases by 30% from 2015 to 2030 [23]. There are a number of chronic diseases affecting the health of perimenopausal females, including cardiovascular diseases, osteoporosis, and cancer. In addition, the reproductive changes of Chinese women especially in developed regions such as Shanghai may also cause a change of the disease spectrum. How to more precisely target high risk population and achieve more efficient health resource allocation is the key to achieve effective prevention and improve women’s health in particular.

There were several limitations of the current study. First, there is no information regarding treatment and histological types, and we could not look into the treatment effect on cancer survival. Unlike the GBD Study which reported comprehensive epidemiological profiles of cancer burden including estimated cancer incidence, mortality, years lived with disability, YLL, and disability-adjusted life-years [17], the current study focused on the total disease burden of MNFGO. Second, PNA is a region in Shanghai and may not be representative of the metropolitan Shanghai. However, it has a proportioned mixture of urban, suburban and rural population, and can reflect more comprehensive situations compared to districts only comprised of urban population [2]. In addition, unlike studies such as the GBD which relied on data from different sources with various qualities to provide best estimates [17], our study is based on complete and accurate population data covering over two decades from the government surveillance system, and high data quality is assured. It is well known that population-based data are crucial to develop and implement prevention and control strategies.

## Conclusion

To summarize, the current study comprehensively analyzed cancer statistics including mortality, YLL due to MNFGOs and their trends in PNA of Shanghai, China during a period of 23 years. Our study is crucial to provide population-based evidence for cancer research, future policy design, and health resource allocation for vulnerable population (women) in China and in other similar cities in the world.

## Supplementary information

**Additional file 1: Figure. S1.** Age composition of the population in Shanghai Pudong New Area from 1995 to 2018.

**Additional file 2: Figure. S2.** Trends of the proportion of ≥65 years age group in the total and female population in Shanghai Pudong New Area; and the capital per GDP in Shanghai and Shanghai Pudong New Area, from 1995 to 2018.

**Additional file 3: Table S1.** Number and proportion of different causes of MNFGO death in each age group in Shanghai PNA, 1995-2018.

**Additional file 4: Figure. S3.** The observed CMR, ASMRW and YLL rate of all MNFGO in different cancer types and age groups in Shanghai Pudong New Area from 1995 to 2018. A, CMR of pathology types; B, ASMRW of pathology types; C, YLL rate of pathology types; D, CMR of age groups; E, YLL rate of age groups. Abbreviations: ASMRW, age-standardized mortality rate by Segi’s world standard population (per 100,000); CMR, crude mortality rate (per 100,000); YLL, years of life lost (per 100,000).

**Additional file 5: Table S2.** Observed values of CMR, ASMRW, YYL and YYL rate in age groupds and the top four MNFGO cancer types in Shanghai PNA, 1995-2018.

**Additional file 6: Table S3.** The increased rates caused by demographic and non-demographic factors and their contribution rates during the period from 1998 to 2018 compared with the CMR of different MNFGO types during 1995–1997 or the period before it in Shanghai PNA.

## Data Availability

The data that support the findings of this study are available from Center for Disease Control and Prevention of the Pudong New Area, Shanghai but restrictions apply to the availability of these data, which were used under license for the current study, therefore are not publicly available. However, data and materials can be obtained from the corresponding author (Dr. Xiaopan Li) under reasonable request with permission from Center for Disease Control and Prevention of the Pudong New Area.

## Abbreviations

95% CI: 95% confidence interval
95% UI: 95% uncertainty interval
APC: Annual percent change
ASDR: Age-standardized death rate
ASMRW: Age-standardized mortality rate by Segi’s world standard population
CDC: Center for Disease Control and Prevention
CMR: Crude mortality rate
DCO: Death certificate only
GBD: GLOBAL Burden of Disease
GDP: Gross domestic product
ICD-9: International Classification of Diseases 9th version
ICD-10: International Classification of Diseases 10th version
MNFGO: Malignant neoplasm of female genital organs
MV: Morphology verified
PNA: Pudong New Area
YLL: Years of life lost
