# Supplementary figures and images for: Years of life lost due to premature death and their trends in people with malignant neoplasm of female genital organs in Shanghai, China during 1995–2018: a population based study
Source: BMC Public Health. 2020 Oct 1;20:1489. doi: 10.1186/s12889-020-09593-6 (PMC7528500; doi:10.1186/s12889-020-09593-6)

A

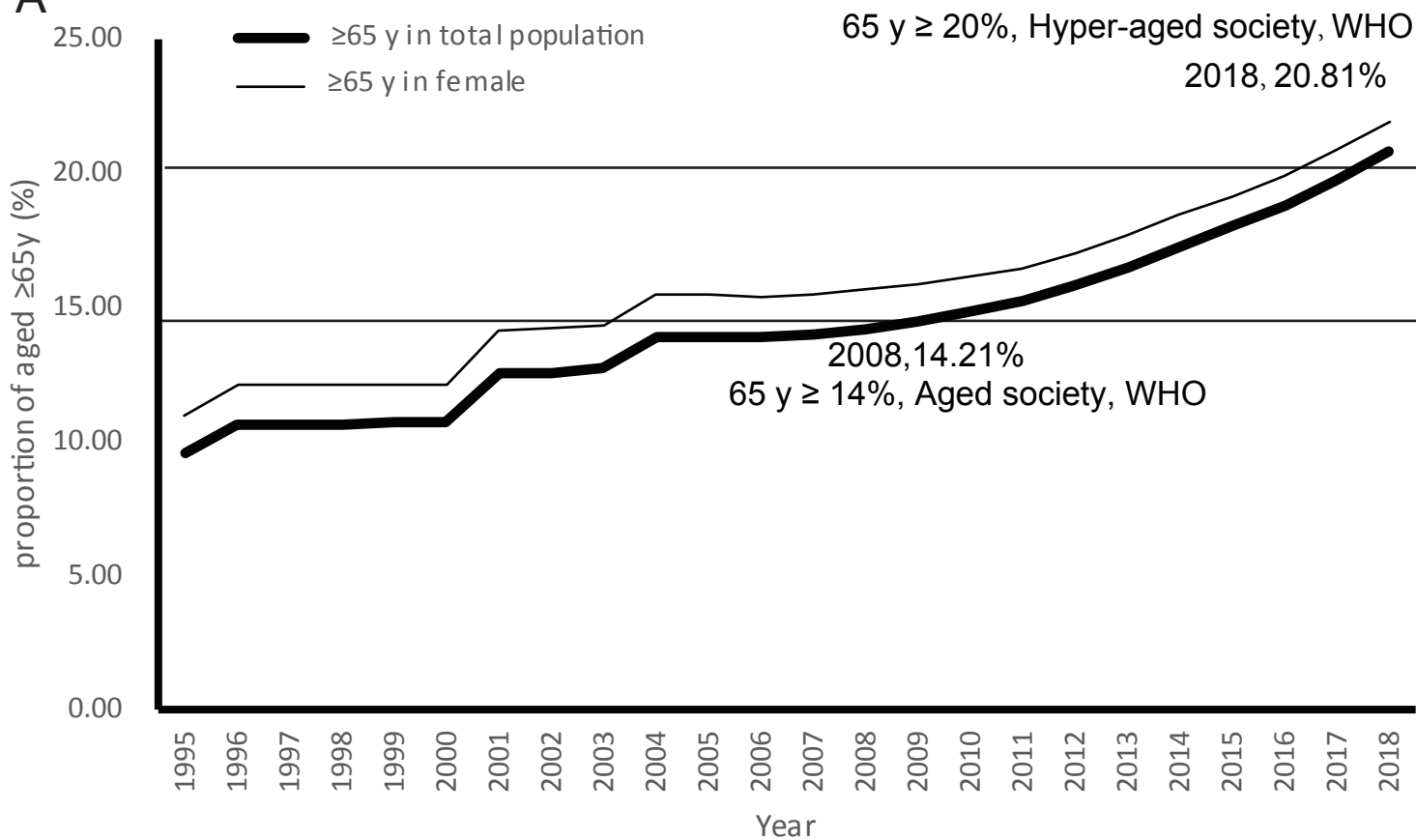

B

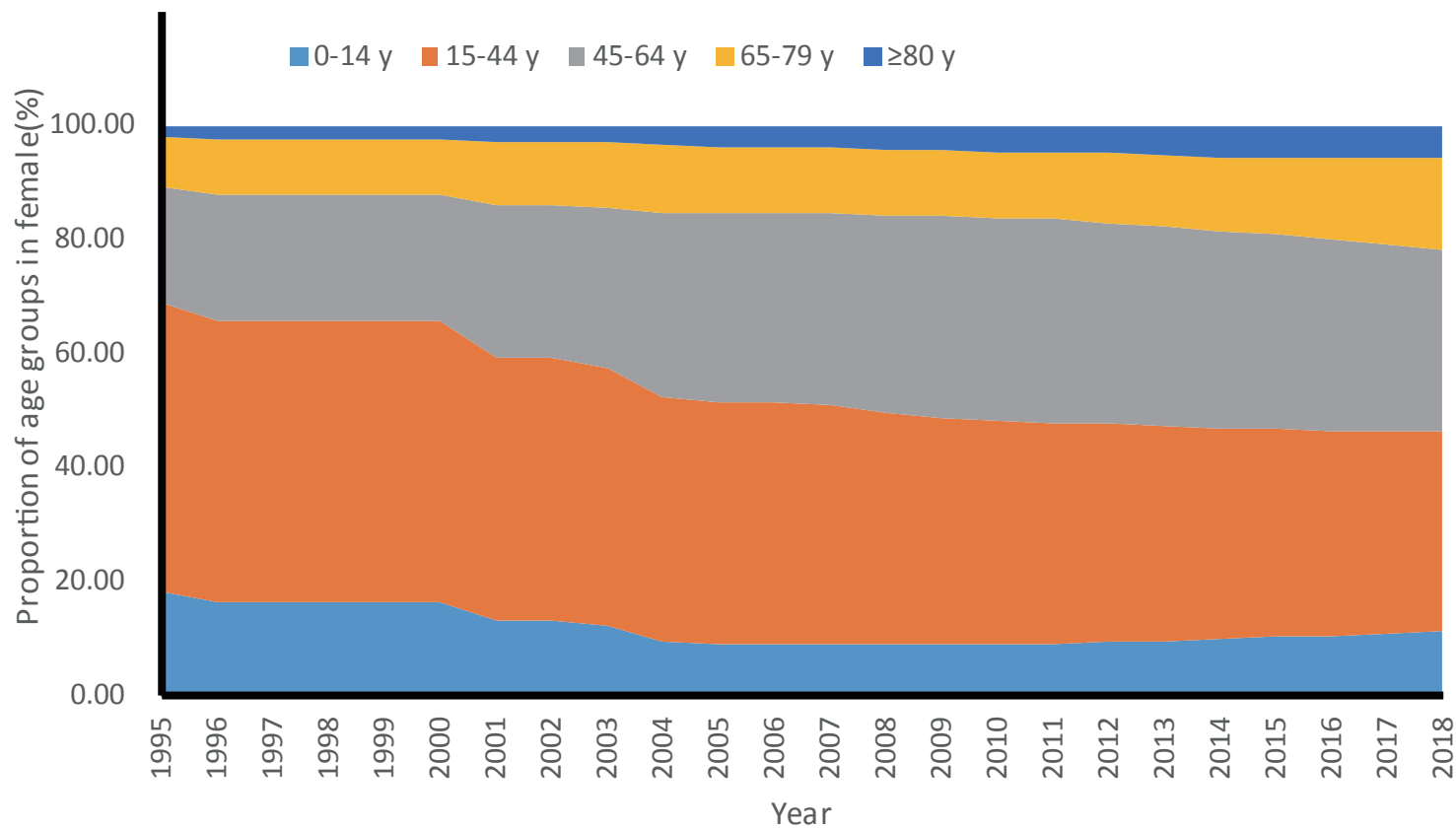

Supplement: Supplementary file 1 — Additional file 1: Figure. S1. Age composition of the population in Shanghai Pudong New Area from 1995 to 2018. [file 12889_2020_9593_MOESM1_ESM.pdf]

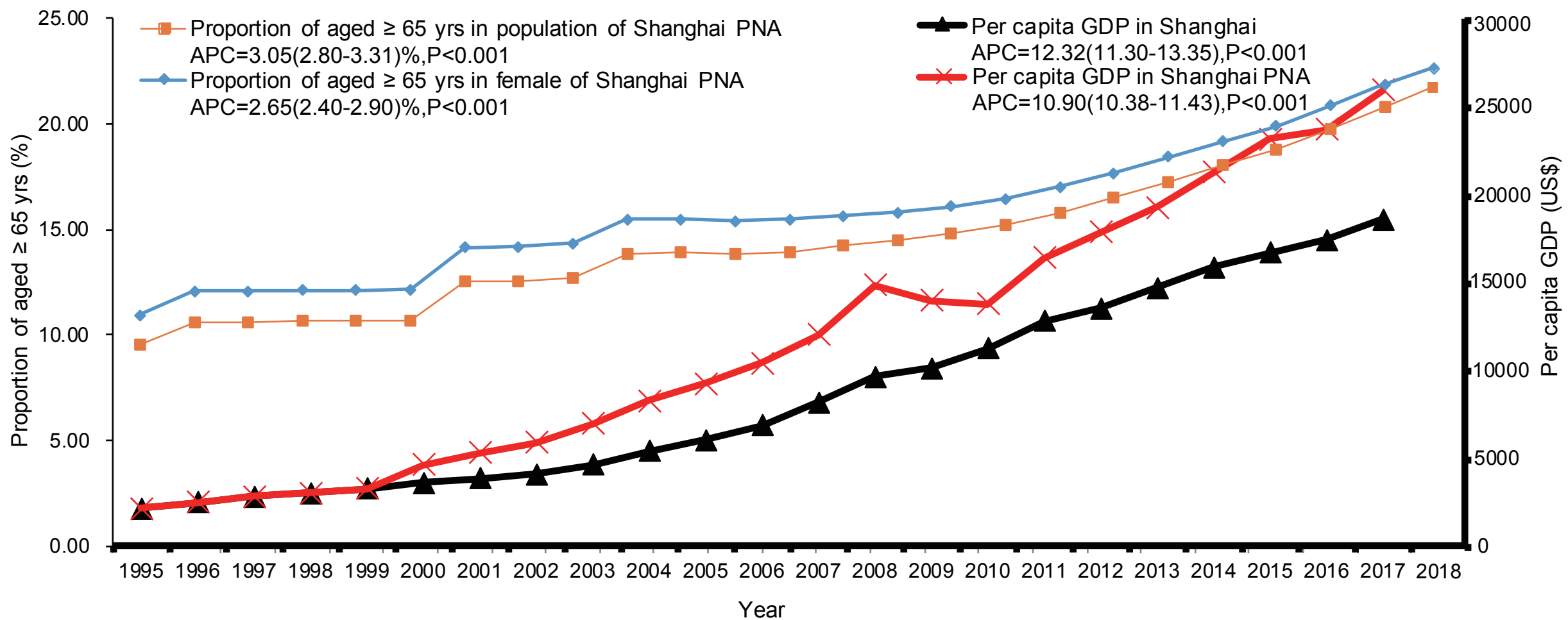

Supplement: Supplementary file 2 — Additional file 2: Figure. S2. Trends of the proportion of ≥65 years age group in the total and female population in Shanghai Pudong New Area; and the capital per GDP in Shanghai and Shanghai Pudong New Area, from 1995 to 2018. [file 12889_2020_9593_MOESM2_ESM.pdf]

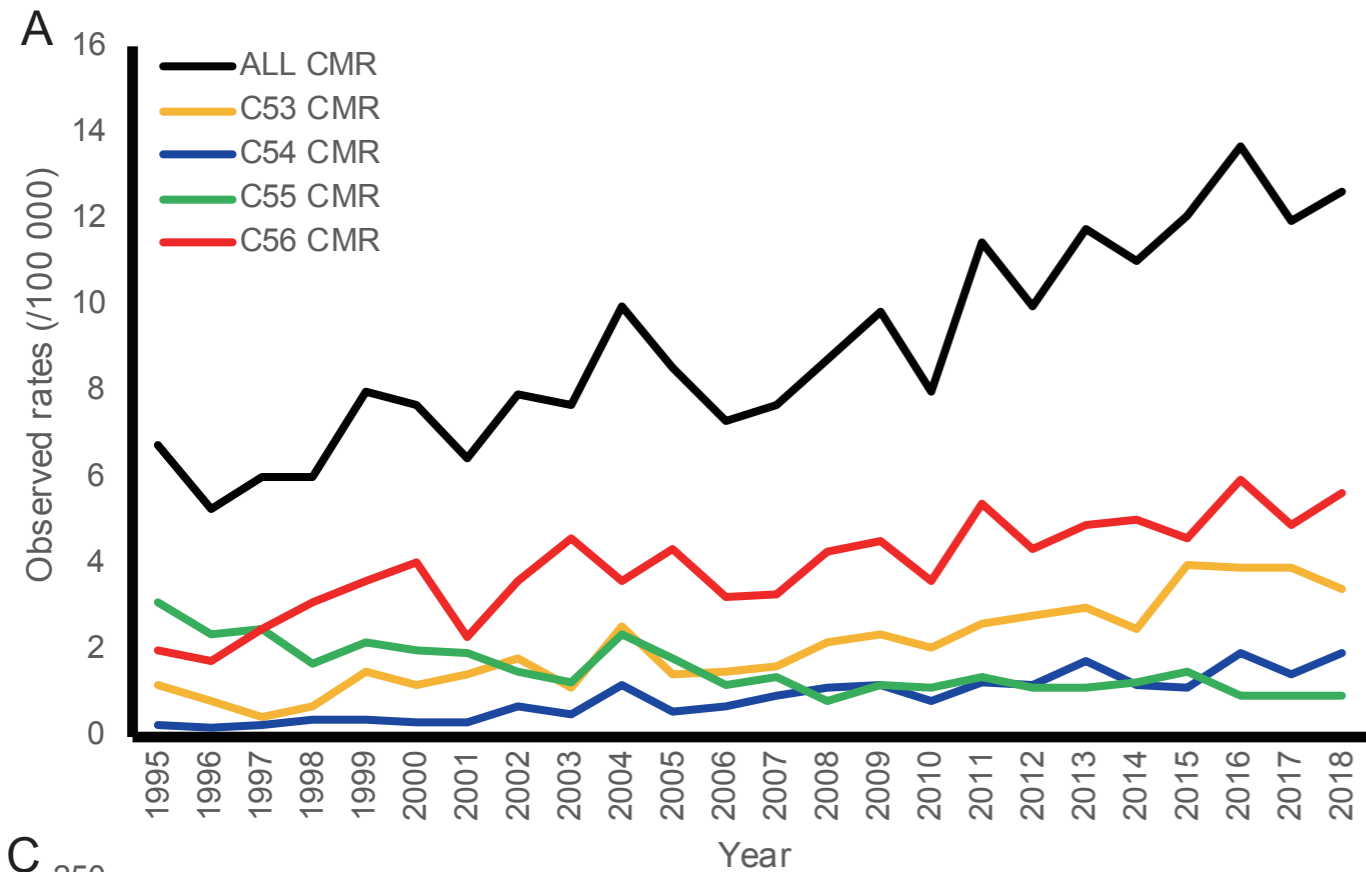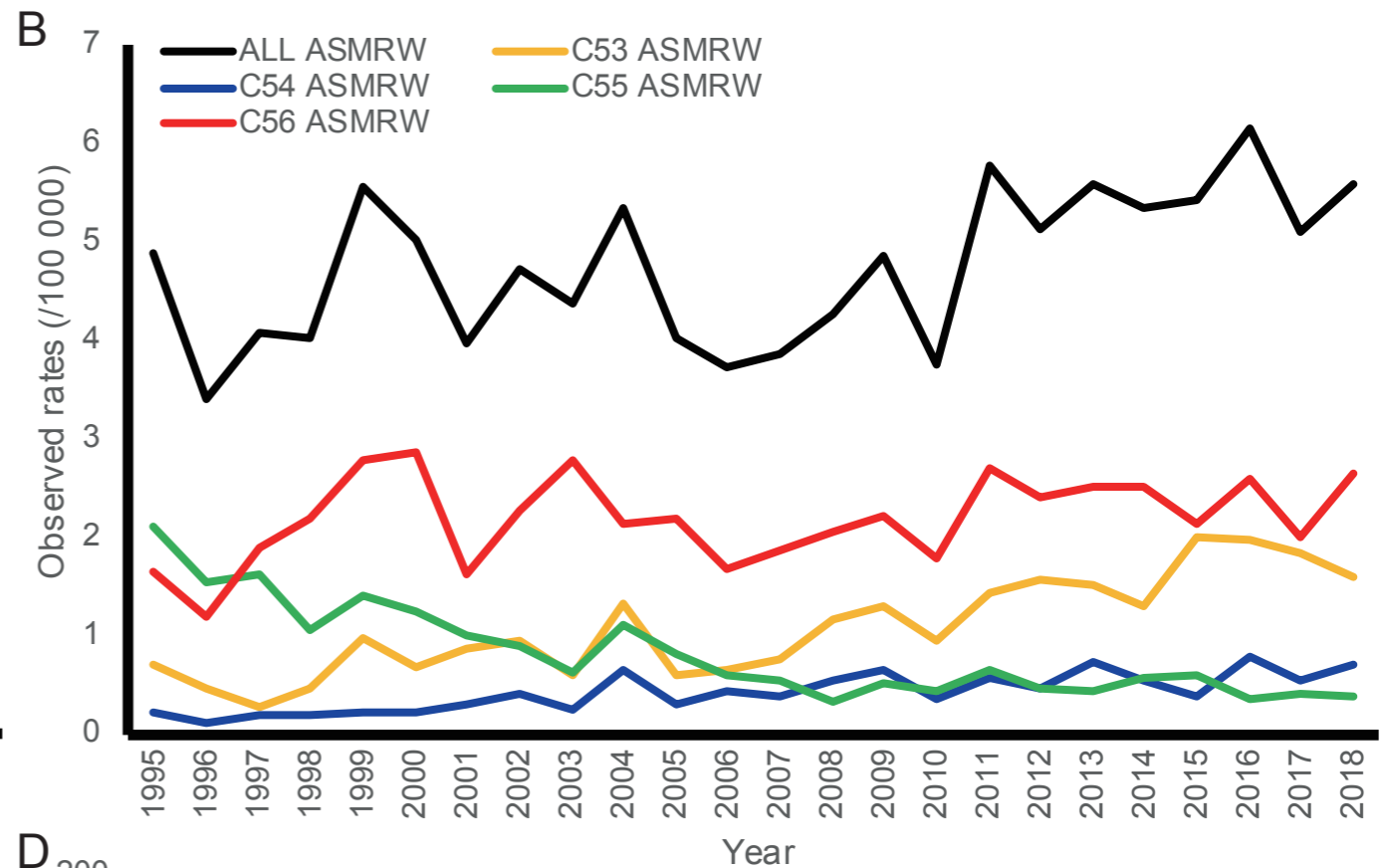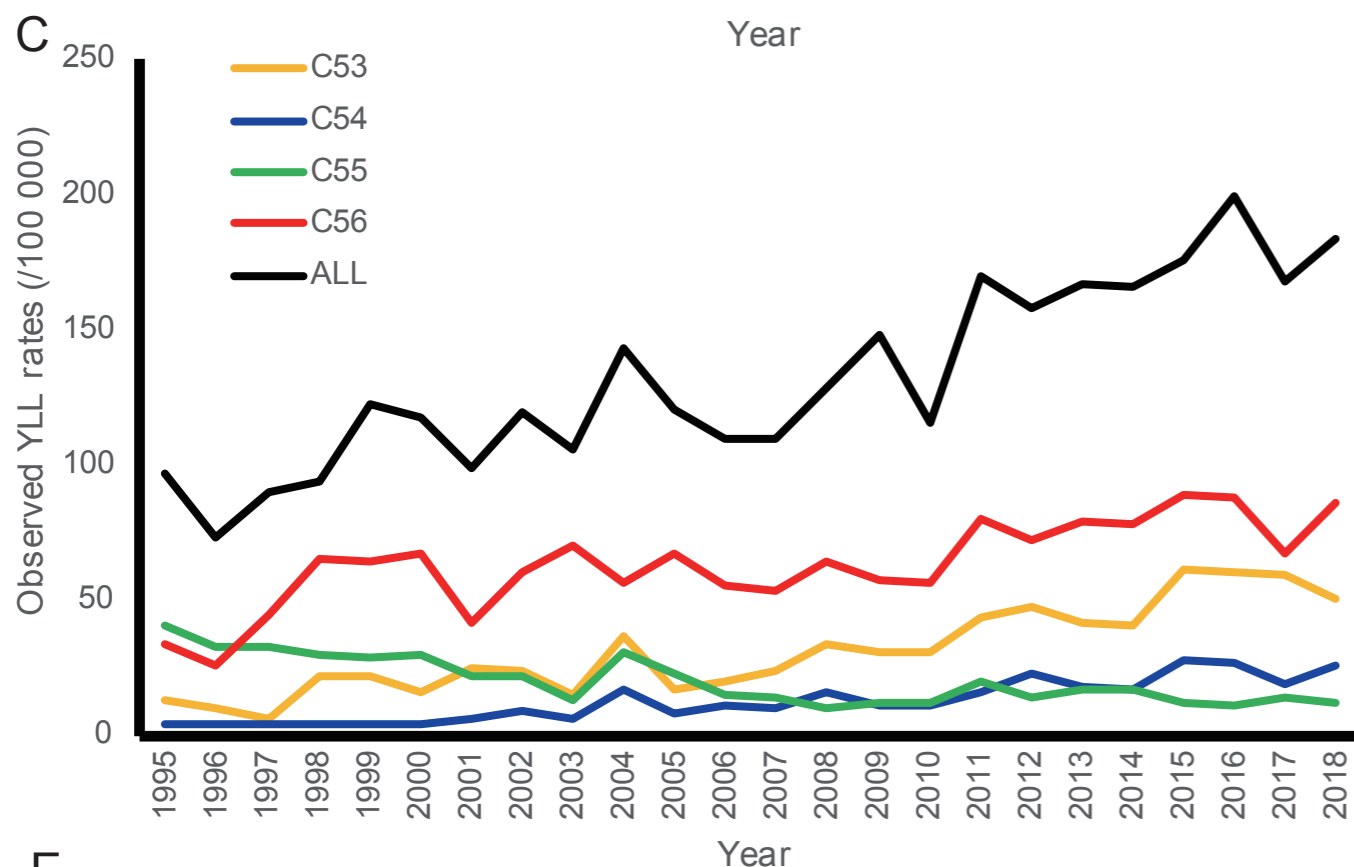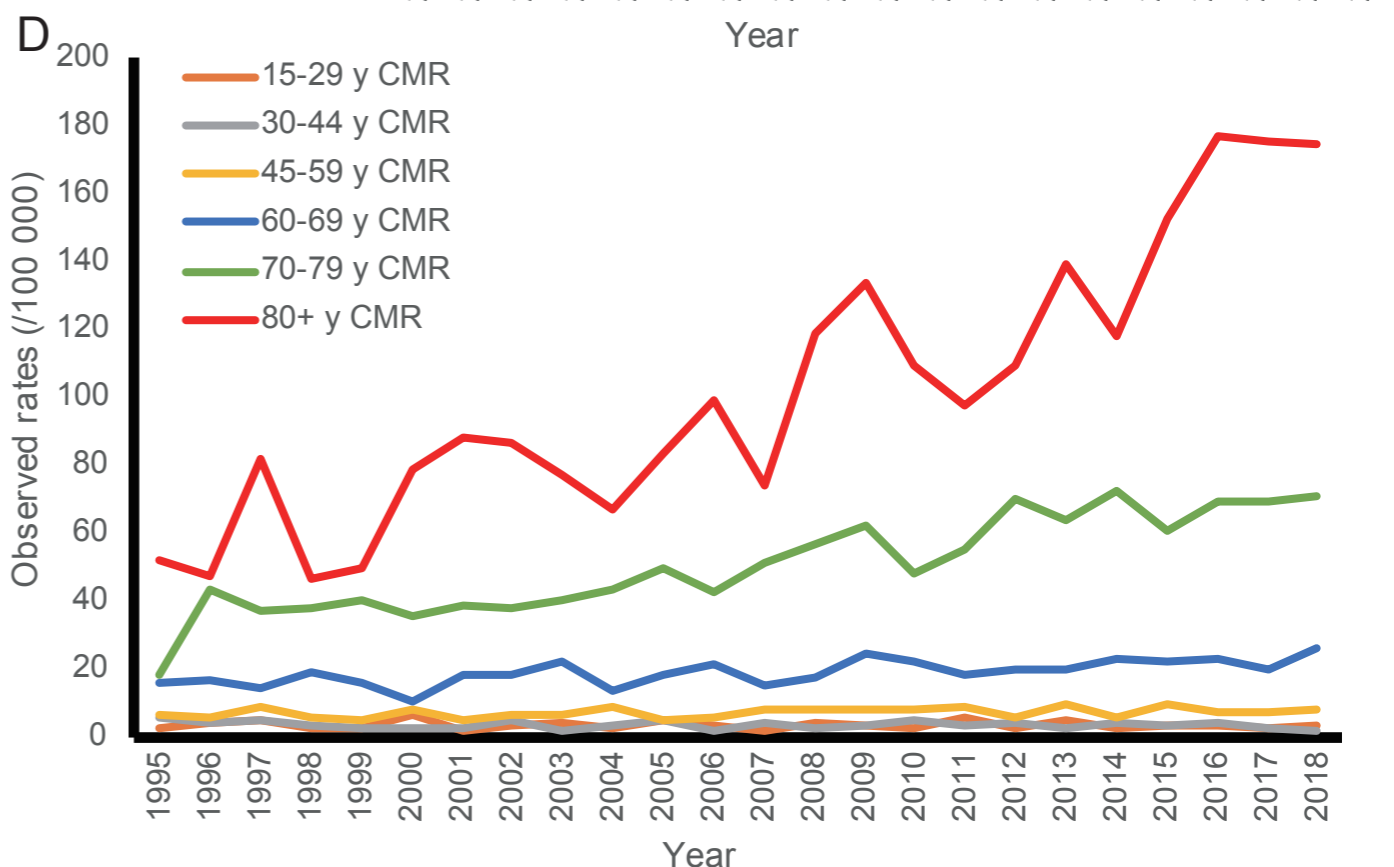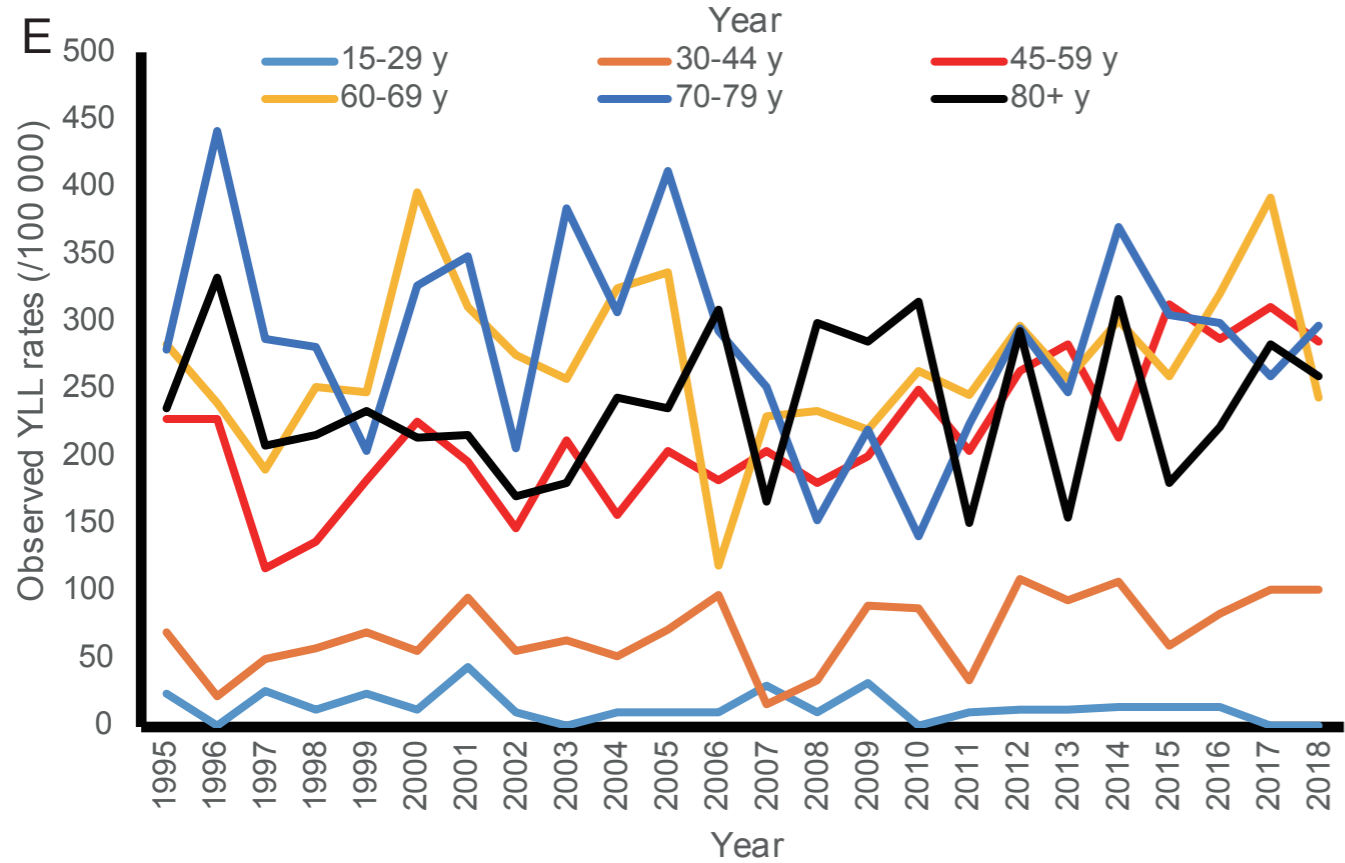

Supplement: Supplementary file 4 — Additional file 4: Figure. S3. The observed CMR, ASMRW and YLL rate of all MNFGO in different cancer types and age groups in Shanghai Pudong New Area from 1995 to 2018. A, CMR of pathology types; B, ASMRW of pathology types; C, YLL rate of pathology types; D, CMR of age groups; E, YLL rate of age groups. Abbreviations: ASMRW, age-standardized mortality rate by Segi’s world standard population (per 100,000); CMR, crude mortality rate (per 100,000); YLL, years of life lost (per 100,000). [file 12889_2020_9593_MOESM4_ESM.pdf]
