# Supplementary material for: Years of life lost due to premature death and their trends in people with malignant neoplasm of female genital organs in Shanghai, China during 1995–2018: a population based study
Source: BMC Public Health. 2020 Oct 1;20:1489. doi: 10.1186/s12889-020-09593-6 (PMC7528500; doi:10.1186/s12889-020-09593-6)
Supplement: Supplementary file 3 — Additional file 3: Table S1. Number and proportion of different causes of MNFGO death in each age group in Shanghai PNA, 1995-2018. [file 12889_2020_9593_MOESM3_ESM.doc]

**Table S1.** Number and proportion of different causes of MNFGO death in each age group in Shanghai PNA, 1995-2018

| **Type** | **Age group** | | | | | | | |
| --- | --- | --- | --- | --- | --- | --- | --- | --- |
|  | **0-14 y** | **15-29 y** | **30-44 y** | **45-59 y** | **60-69 y** | **70-79 y** | **80+ y** | **Total** |
| **Vulva cancer (C51)** | 0 (0) | 0 (0) | 0 (0) | 6 (0.70) | 9 (1.37) | 23 (3.92) | 33 (6.25) | 71 (2.47) |
| **Vagina cancer (C52)** | 0 (0) | 0 (0) | 2 (0.93) | 6 (0.70) | 12 (1.82) | 11 (1.88) | 9 (1.70) | 40 (1.39) |
| **Cervix uteri cancer (C53)** | 0 (0) | 8 (27.59) | 96 (44.86) | 199 (23.33) | 103 (15.65) | 122 (20.82) | 149 (28.22) | 677 (23.60) |
| **Corpus uteri cancer (C54)** | 0 (0) | 1 (3.45) | 8 (3.74) | 71 (8.32) | 91 (13.83) | 60 (10.24) | 62 (11.74) | 293 (10.21) |
| **Uterus unspecified cancer (C55)** | 0 (0) | 1 (3.45) | 25 (11.68) | 104 (12.19) | 101 (15.35) | 120 (20.48) | 120 (22.73) | 471 (16.42) |
| **Ovary cancer (C56)** | 1 (100) | 18 (62.07) | 82 (38.32) | 453 (53.11) | 319 (48.48) | 237 (40.44) | 150 (28.41) | 1260 (43.92) |
| **Other female genital organs cancer (C57)** | 0 (0) | 0 (0) | 1 (0.47) | 14 (1.64) | 23 (3.50) | 13 (2.22) | 5 (0.95) | 56 (1.95) |
| **Placenta cancer (C58)** | 0 (0) | 1 (3.45) | 0 (0) | 0 (0) | 0 (0) | 0 (0) | 0 (0) | 1 (0.03) |
| **Total** | 1 | 29 | 214 | 853 | 658 | 586 | 528 | 2869 |

MNFGO, malignant neoplasm of female genital organs; PNA, Pudong New Area.
